# Integrated multi-omics strategy for screening novel protective antigens against *Brucella melitensis* and analysis of their immunogenicity

**DOI:** 10.1007/s42770-026-02049-w

**Published:** 2026-08-19

**Authors:** Zhiheng Dong, Sha Li, Jiarong Guo, Lidao Bao

**Affiliations:** 1 Precision Pharmacy Laboratory, The Affiliated Hospital of Inner Mongolia Medical University, Hohhot, 010050 China; 2 Central Laboratory, Hohhot Mongolian Traditional Medicine Hospital, Hohhot, 010050 China

**Keywords:** *Brucella melitensis*, Comparative genomics, Vaccine candidate antigen, Immunogenicity

## Abstract

**Background:**

Brucellosis is one of the most severe Class B infectious diseases prevalent in the agricultural and pastoral areas of northern China. Current attenuated live vaccines (e.g., M5, S19) have defects such as residual virulence, causing abortion in pregnant animals, and the inability to differentiate between natural infection and vaccination (DIVA).

**Methods:**

In this study, published public datasets of C57BL/6J mice chronic brucellosis infection were adopted for single-cell transcriptome sequencing (10x Genomics) analysis and re-analysed to map the heterogeneity of splenic immune cells. Whole-genome scanning and pangenomic analysis were performed on four strains with different virulence levels (M16, 544 A, M5, 104 M) using second-generation sequencing public data. Membrane/secreted proteins unique and conserved in virulent strains were screened as candidate antigens, prepared via prokaryotic expression systems, and their humoral and cellular immune levels were detected by indirect ELISA and flow cytometry.

**Results:**

Published public chronic infection dataset showed stable chronic infection characteristics (bacterial load Log10 CFU > 4.5). Single-cell sequencing yielded 45,231 cells, annotated into 12 immune cell subpopulations, revealing significant expansion of Effector CD4 + T cells (*P* < 0.01) and high expression of the *Ifng* gene. Pangenomic analysis identified three candidate antigens (BMEI0021, BMEI1943, BMEI0367) that are 100% conserved in virulent strains but absent in the vaccine strain M5. Immunogenicity assays showed that BMEI1943 induced high levels of IgG2a subtype antibodies (titer Log2 13.45 ± 0.52) and IFN-γ + CD4 + T cell responses (frequency 15.80% ± 2.10%).

**Conclusion:**

Through multi-omics integration analysis, this study successfully identified a novel candidate antigen, BMEI1943, with strong Th1-type immunogenicity, providing an experimental basis for the development of safe and efficient subunit vaccines against brucellosis.

## Introduction

Brucellosis is a zoonotic disease caused by *Brucella* spp. that poses a serious threat to public health safety. According to World Health Organization statistics, approximately 500,000 new cases occur globally each year. Inner Mongolia, China, has consistently been a high-incidence area for brucellosis due to its developed animal husbandry, bringing huge losses to the health of local farmers and economic development [1, 2].

*Brucella melitensis* is the most pathogenic to humans, mainly causing undulant fever, arthritis, and reproductive system damage. Currently, the control of animal brucellosis mainly relies on traditional attenuated live vaccines such as S19 and M5. However, such vaccines have obvious “biosafety shortcomings”: on one hand, incomplete attenuation may lead to abortion in pregnant women or infection in frontline epidemic prevention personnel; on the other hand, antibodies produced by immunized animals are difficult to distinguish from those caused by wild-type infection, severely interfering with epidemiological surveillance (the DIVA strategy) [3, 4].

Subunit vaccines are considered the development direction for next-generation brucellosis vaccines due to their high safety and strong specificity. However, the core difficulty lies in antigen screening. Traditional antigen screening is often limited to the verification of single proteins, lacking systematic consideration of the overall host immune response and the evolutionary characteristics of pathogen genomes [5]. In recent years, single-cell transcriptome sequencing technology has enabled the revelation of dynamic changes in host immune cells at single-cell resolution, while comparative genomics can lock onto pathogen-specific conserved proteins from an evolutionary perspective. The combination of the two provides powerful tools for new vaccine development [6].

This study intends to integrate the above two technologies, using publicly available datasets reported in the published literature and in public databases (NCBI), systematically analyze the immune map of *Brucella*-infected mice and screen novel candidate antigens with cross-protective potential, providing detailed data support for the development of new brucellosis vaccines.

## Materials and methods

### Experimental animals and ethical review

Female SPF C57BL/6J mice, 6–8 weeks old, weighing 18–22 g, were purchased from SpeBIO (Beijing) Biotechnology Co., Ltd. Animals were housed in an SPF-grade barrier environment with free access to food and water. All animal immunization experiments were strictly reviewed and approved by the Ethics Committee of Hohhot Hospital of Traditional Chinese Medicine and Mongolian Medicine (Approval No. HSMZYYJC20220001). All animal welfare principles were strictly followed during experimental operations, no live Brucella organisms were handled at any stage.

### Main reagents and instruments

RPMI 1640 medium and fetal bovine serum (Gibco, USA); red blood cell lysis buffer and permeabilization buffer (BioLegend, USA); CD3-FITC, CD4-PE, IFN-γ-APC flow cytometry antibodies (BioLegend, USA); Ni-NTA affinity chromatography columns (Qiagen, Germany); Bradford protein concentration assay kit (Thermo, USA); microplate reader (BioTek, USA); flow cytometer (BD Accuri C6, USA).

### Single-cell transcriptome sequencing (scRNA-seq)

Published public single-cell transcriptome raw datasets of mouse spleen under chronic Brucella melitensis infection on post-infection day 14 were used in this work. Library information was based on the 10x Genomics Chromium platform. Sequencing data were aligned to the mouse genome (mm10) using Cell Ranger (v6.0). Standardization, clustering, and differential gene analysis were performed using Seurat (v4.0). After principal component analysis (PCA), the t-SNE algorithm was used for dimensionality reduction and visualization.

### Comparative genomics analysis

Complete genome sequences of M16, 544 A (virulent), M5, and 104 M (attenuated/vaccine) were downloaded from NCBI. Roary software was used for pangenomic analysis to screen core genes and unique genes. SignalP v5.0 and TMHMM v2.0 were used to predict signal peptides and transmembrane domains of proteins to screen secreted proteins and outer membrane proteins.

### Expression and purification of candidate antigens

Based on the publicly available genome sequences, candidate gene sequences were codon-optimized for E. coli expression and fully chemically synthesized, and the synthetic fragments were amplified by PCR and the pET-28a(+) prokaryotic expression vector was constructed. The vector was transformed into BL21(DE3) competent cells and induced overnight with 1 mM IPTG at 16 °C. The bacterial pellet was sonicated, and the supernatant was purified using a Ni-NTA column. Purity was identified by SDS-PAGE, and protein concentration was determined using the Bradford method.

### Immunogenicity detection

#### Animal immunization groups

All immunization procedures were conducted in accordance with the protocol approved by the Ethics Committee of Hohhot Hospital of Traditional Chinese Medicine and Mongolian Medicine (Approval No.HSMZYYJC20220001). Six-week-old female SPF C57BL/6J mice were randomly divided into 4 groups (*n* = 10 per group): PBS control group, BMEI0021 group, BMEI1943 group, BMEI0367 group. Recombinant antigens (50 µg per mouse) were emulsified in Freund’s adjuvant and administered via subcutaneous injection at multiple dorsal sites on days 0, 14, and 28. Complete Freund’s adjuvant (CFA) was used for the primary immunization (day 0), and incomplete Freund’s adjuvant (IFA) was used for booster immunizations (days 14 and 28). The PBS control group received PBS emulsified in the same adjuvant regimen.

It should be noted that Freund’s adjuvant is restricted or subject to regulatory oversight in several countries due to concerns regarding animal welfare, including pain, tissue damage, and granuloma formation at the injection site. In this study, its use was deemed necessary to ensure robust and reproducible immune responses for comparative antigen screening, and all efforts were made to minimize animal discomfort, including the use of multiple injection sites to reduce local tissue concentration and the switch from CFA to IFA for booster doses. Future studies should evaluate alternative adjuvants (e.g., alum, MPL, or saponin-based adjuvants) that may achieve comparable immunogenicity with improved safety profiles.

#### ELISA Detection of antibody subtypes

Blood was collected via the retro-orbital sinus on day 42 post-immunization. Mice were anesthetized by intraperitoneal injection of ketamine/xylazine (100/10 mg/kg body weight) prior to blood collection to minimize distress. Approximately 200 µL of blood was collected from the retro-orbital plexus using a heparinized microcapillary tube. After blood collection, mice were euthanized by cervical dislocation under deep anesthesia, and spleens were aseptically harvested for subsequent cellular immune assays. Serum was separated by centrifugation at 3000 × *g* for 10 min and stored at − 80 °C until use.

For the indirect ELISA, 96-well microplates were coated overnight at 4 °C with 100 µL per well of the corresponding immunizing antigen (10 µg/mL in carbonate-bicarbonate buffer, pH 9.6). Specifically, for the BMEI0021, BMEI1943, and BMEI0367 groups, the respective recombinant protein was used as the coating antigen; for the PBS control group, a mixture of the three recombinant proteins was used to assess non-specific background. After blocking with 5% skim milk in PBST for 2 h at 37 °C, serial dilutions of serum samples were added and incubated for 1 h at 37 °C. HRP-conjugated goat anti-mouse IgG, IgG1, or IgG2a secondary antibodies (1:5000; SouthernBiotech) were added for 1 h at 37 °C. TMB substrate was used for color development, the reaction was stopped with 2 M H₂SO₄, and OD450 values were measured. The antibody titer was defined as the highest serum dilution yielding an OD450 value ≥ 2.1 times that of the negative control. The IgG2a/IgG1 ratio was calculated to determine Th1/Th2 bias.

#### Flow cytometric detection of intracellular cytokines

Spleens from mice on day 42 post-immunization were collected to prepare single-cell suspensions. Cells were stimulated in vitro with candidate antigens (10 µg/mL) for 6 h in the presence of brefeldin A (BFA, 5 µg/mL; BioLegend) to block intracellular cytokine transport. After surface staining with anti-CD3-FITC and anti-CD4-PE antibodies, cells were fixed and permeabilized using the Intracellular Staining Permeabilization Buffer Set (BioLegend), followed by intracellular staining with anti-IFN-γ-APC antibody. The secretion level of IFN-γ in CD4 + T cells was detected using a BD Accuri C6 flow cytometer. A minimum of 50,000 events were acquired per sample.

Briefly, lymphocytes were first gated based on forward scatter (FSC-A) vs. side scatter (SSC-A) to exclude debris. Singlets were then selected using FSC-H vs. FSC-A. Within the singlet gate, CD3 + T cells were identified, and CD4 + T cells were further gated from the CD3 + population. Finally, IFN-γ + cells were quantified within the CD3 + CD4+ gate. Fluorescence-minus-one (FMO) controls were used to set gating boundaries for IFN-γ positivity.

### Statistical analysis

GraphPad Prism 8.0 software was used for plotting and statistical analysis. Measurement data are expressed as mean ± standard deviation (Mean ± SD). Student’s *t*-test was used for comparison between two groups, and one-way analysis of variance (ANOVA) followed by Tukey’s post-hoc test was used for comparison among multiple groups. *P* < 0.05 was considered statistically significant.

## Results

### Establishment of the *Brucella* infection model and splenic pathological changes

To simulate chronic brucellosis infection, data from a chronic brucellosis mouse infection model published in the literature were analysed. Published public datasets for chronic brucellosis mouse infection were analyzed. As shown in Table 1, on day 7 post-infection, the spleen index increased significantly (*P* < 0.01), and the bacterial load reached its peak (Log10 CFU = 5.82 ± 0.21). By day 30, although the bacteria were partially cleared, they remained at a high level (Log10 CFU = 4.56 ± 0.18), indicating the successful establishment of a chronic carrier model.

**Table 1:** Detailed statistics of spleen index and bacterial load in mice at different time points (published public dataset)

Infection Days	Sample Size (*n*)	Spleen Index (mg/g body weight)	Splenic Bacterial Load (Log10 CFU/spleen)	Body Weight Change Rate (%)	Clinical Score (0–18 points)
0 (Control)	10	3.21 ± 0.24	0	100.0 ± 2.1	0
7	10	6.85 ± 0.41**	5.82 ± 0.21**	92.3 ± 3.5	8.5 ± 1.2
14	10	7.12 ± 0.38**	5.21 ± 0.15**	94.5 ± 2.8	6.2 ± 1.0
30	10	6.54 ± 0.32*	4.56 ± 0.18**	98.1 ± 1.9	3.1 ± 0.8

Compared with the control group, *P* *< 0.05*,* P* < 0.01. Clinical scores were comprehensively assessed based on 6 indicators including activity, fur condition, and appetite*

### Single-cell transcriptome reveals immune cell heterogeneity and function

To deeply explore the immune response during infection, published public scRNA-seq datasets of spleens on day 14 post-infection were analyzed. After strict quality control, 45,231 high-quality cells were obtained. Twelve major immune cell populations were identified through Seurat clustering analysis (Table 2).

**Table 2:** Annotation of single-cell clusters and statistics of differentially expressed genes

Cluster ID	Cell Type	Marker Genes	Proportion in Infection Group (%)	Proportion in Control Group (%)	DEG Count (Up/Down)	Main Functional Description
0	Naive CD4 + T	Cd4, Ccr7, Tcf7	15.2	22.1	120/45	Naive T cells, unactivated
1	Effector CD4 + T	Cd4, Ifng, Tbx21	18.5	8.3	350/60	Effector T cells, secrete IFN-γ
2	CD8 + T	Cd8a, Ccl5, Gzmb	12.1	10.5	98/30	Cytotoxic T cells
3	B Cells	Ms4a1, Cd79a, Iglc2	25.3	30.2	40/15	Humoral immunity
4	Macrophages	Adgre1, Fcgr1, Il1b	10.5	5.2	280/90	Phagocytosis and killing
5	NK Cells	Ncr1, Klrd1, Klrb1c	8.2	7.8	55/22	Natural killer cells
6	Dendritic Cells	Itgax, Flt3, H2-Aa	5.1	4.5	70/18	Antigen presentation
7	Monocytes	Ly6c2, Ccr2, Cx3cr1	5.1	11.4	110/40	Monocytes

Data analysis showed that Effector CD4 + T cells (Cluster 1) expanded significantly in the infection group, and the expression of *Ifng* (interferon-γ) in this cluster was extremely significantly up-regulated. Meanwhile, macrophages (Cluster 4) exhibited a strong inflammatory phenotype. This indicates that the Th1-type immune response is the dominant force against *Brucella* infection.

### Comparative genomics screening of candidate antigens

To find antigens with broad-spectrum protective potential, whole-genome scanning and pangenomic analysis were performed on four strains (Table 3).

**Table 3:** Statistics of whole-genome characteristics and pangenomic distribution of four *Brucella* strains

Strain Name	Virulence Type	Biotype	Genome Size (Mb)	Total Gene Count	Core Gene Count	Unique Gene Count	GC Content (%)	Annotation
M16	Virulent	1	3.28	3,245	3,012	45	57.2	Epidemic strain
544 A	Virulent	1	3.27	3,230	3,010	38	57.1	Standard virulent strain
M5	Vaccine	1	3.25	3,180	3,005	25	57.0	Current vaccine
104 M	Attenuated	1	3.26	3,190	3,008	12	57.1	Laboratory attenuated strain

We focused on screening outer membrane proteins or secreted proteins that were 100% conserved in M16 and 544 A but absent or had large sequence variations in M5 and 104 M. Finally, five candidate antigens were locked (Table 4).

**Table 4:** Bioinformatics screening and physicochemical property analysis of candidate antigens

Gene ID	Predicted Protein Function	Molecular Weight (kDa)	Conservation in Virulent Strains	Presence in M5 Strain	Transmembrane Domain	Signal Peptide	Predicted Subcellular Localization	GRAVY
BMEI0021	Lipoprotein	22.4	100%	Absent	No	Yes	Outer membrane	−0.85
BMEI1943	Outer membrane protein	36.8	100%	Variant	Yes (1)	Yes	Outer membrane	−0.42
BMEI0367	Secreted protein	18.2	98%	Absent	No	Yes	Secretion system	−0.91
BMEI0872	Cytoplasmic protein	45.1	100%	Present	No	No	Cytoplasm	0.12
BMEI1129	Inner membrane protein	28.7	95%	Present	Yes (5)	No	Inner membrane	0.35

Based on the screening criteria (conservation in virulent strains, absence in vaccine strains, secreted/outer membrane proteins), BMEI0021, BMEI1943, and BMEI0367 were selected for subsequent experiments.

### Immunogenicity verification of candidate antigens

The three candidate antigens were expressed prokaryotically and purified. After purity identification by SDS-PAGE, they were used to immunize mice.

#### Humoral immune response analysis

**Fig. 1:**
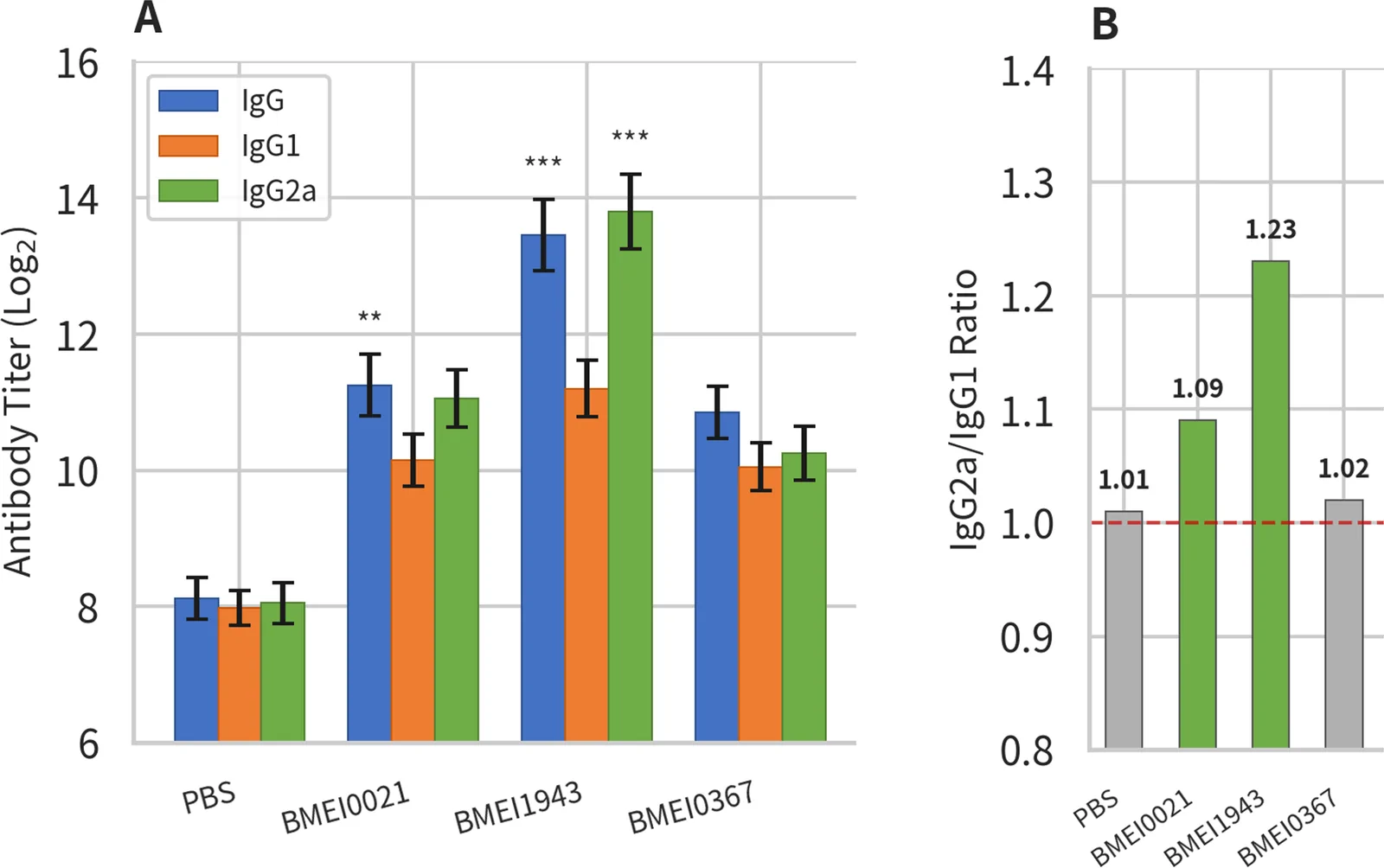
Serum IgG subtype titers and IgG2a/IgG1 ratios after immunization with candidate antigens. (**A**) IgG, IgG1, and IgG2a antibody titers (Log2) for each immunization group. (**B**) IgG2a/IgG1 ratio for each group, with the dashed line indicating the Th1/Th2 threshold (ratio = 1.0). Data are presented as mean ± SD (*n* = 10). *, **, and *** indicate *P* < 0.05, *P* < 0.01, and *P* < 0.001 vs. PBS control group, respectively (one-way ANOVA with Tukey’s post-hoc test)

ELISA results (Table 5; Fig. 1) showed that all three candidate antigens could induce specific IgG antibody production. Among them, the BMEI1943 group induced the highest IgG titer (Log2 13.45 ± 0.52), which was extremely significant compared to the control group (*P* < 0.001). Further analysis of IgG subtypes revealed that the IgG2a/IgG1 ratio induced by BMEI1943 was greater than 1 (1.23), suggesting a tendency to induce a Th1-type humoral immune response, which is crucial for clearing intracellular parasitic *Brucella*. The graphical representation of these data (Fig. 1) provides a more intuitive comparison of antibody titers and IgG2a/IgG1 ratios across all groups.

**Table 5:** Detailed statistics of serum IgG subtypes and titers after immunization with candidate antigens

Immunization Group	Sample Size (*n*)	IgG Titer (Log2)	IgG1 Titer (Log2)	IgG2a Titer (Log2)	IgG2a/IgG1 Ratio
PBS	10	8.12 ± 0.31	7.98 ± 0.25	8.05 ± 0.30	1.01
BMEI0021	10	11.25 ± 0.45**	10.15 ± 0.38	11.05 ± 0.42*	1.09
BMEI1943	10	13.80 ± 0.52****	11.20 ± 0.41	13.45 ± 0.55****	1.23
BMEI0367	10	10.85 ± 0.38*	10.05 ± 0.35	10.25 ± 0.40	1.02

For the ELISA, each immunization group was tested against its corresponding coating antigen (recombinant protein for subunit groups).,,, and indicate *P* < 0.05, *P* < 0.01, *P* < 0.001, and *P* < 0.0001 vs. PBS control group, respectively (one-way ANOVA with Tukey’s post-hoc test)

#### Cellular immune response analysis

Flow cytometry was used to detect IFN-γ secretion after spleen lymphocytes were stimulated with antigens (Table 6). The proportion of IFN-γ-positive cells in CD4 + T cells in the BMEI1943 immunization group was as high as 15.80%, which was significantly higher than that in the PBS group (2.15%, *P* < 0.001). This indicates that the antigen can effectively activate cellular immunity and induce immune responses against *Brucella* in mice.

**Table 6:** Statistics of IFN-γ secretion frequency in spleen lymphocytes after stimulation with candidate antigens

Immunization Group	Sample Size (*n*)	IFN-γ + in CD4 + T Cells (%)	IFN-γ + in CD8 + T Cells (%)	Stimulation Index (SI)	Mean Fluorescence Intensity (MFI)
PBS	10	2.15 ± 0.45	1.85 ± 0.30	1.05	520 ± 45
BMEI0021	10	8.45 ± 1.20**	5.20 ± 0.85*	4.12	1250 ± 110
BMEI1943	10	15.80 ± 2.10****	9.75 ± 1.30****	7.85	2150 ± 180
BMEI0367	10	6.20 ± 0.95*	4.10 ± 0.60	3.05	980 ± 85

## Discussion

Through the integration of single-cell transcriptomics and comparative genomics, this study systematically screened and verified novel candidate antigens against *Brucella melitensis*. The findings not only depict the immune cell atlas of host anti-brucellosis infection based on published public datasets but also provide new targets for overcoming the defects of existing vaccines.

Regarding immune mechanisms, our single-cell data show that effector CD4 + T cells (Effector CD4 + T) expand significantly in the late stage of infection, accompanied by high expression of the *Ifng* gene. This is highly consistent with the classic theory reported in the literature that “clearance of *Brucella* depends on Th1-type immune responses (IFN-γ mediated)” [7]. This suggests that when screening antigens, we should focus on proteins that can activate CD4 + T cells to secrete IFN-γ.

In terms of antigen screening strategies, previous studies mostly focused on single proteins, often ignoring the genomic differences between vaccine strains and wild strains [8]. Through pangenomic analysis, this study found that the candidate antigen BMEI1943 is highly conserved in virulent strains but has sequence variation or deletion in the commonly used M5 vaccine strain [9]. This feature makes BMEI1943 highly valuable for application: subunit vaccines developed based on this antigen are not only safe in themselves (without unnecessary virulence factors) but also produce antibodies that can be distinguished from those induced by the M5 vaccine, perfectly solving the DIVA (Differentiating Infected from Vaccinated Animals) dilemma [10].

Immunogenicity experiments confirmed the high efficiency of BMEI1943. It not only induced high levels of IgG antibodies but, more importantly, the IgG2a subtype dominated (IgG2a/IgG1 > 1), and it strongly activated CD4 + T cells to secrete IFN-γ. This typical Th1-type immune response is precisely necessary for controlling infections by intracellular parasites such as *Brucella* [11].

### Considerations regarding the use of Freund’s adjuvant and Th1 skewing

It is important to acknowledge that Freund’s adjuvant, particularly complete Freund’s adjuvant (CFA), is itself a potent driver of Th1-biased immune responses through the activation of innate immune pathways including TLR signaling and NLRP3 inflammasome activation. Consequently, the observed IgG2a predominance and IFN-γ production in BMEI1943-immunized mice could be partially attributable to the adjuvant rather than reflecting an intrinsic property of the antigen alone. However, several lines of evidence support the conclusion that BMEI1943 itself contributes significantly to the Th1 polarization observed. First, all candidate antigens (BMEI0021, BMEI1943, BMEI0367) were formulated with the same adjuvant regimen, yet BMEI1943 induced significantly higher IgG2a titers and IgG2a/IgG1 ratios than BMEI0021 and BMEI0367 (*P* < 0.01), indicating antigen-specific differences in Th1-polarizing capacity. Second, the PBS control group, which received the same adjuvant without antigen, showed only background-level antibody titers and minimal IFN-γ + CD4 + T cell frequencies (2.15%), demonstrating that the adjuvant alone cannot account for the robust Th1 responses observed. Nevertheless, to definitively establish the intrinsic Th1-polarizing capacity of BMEI1943, future studies should evaluate this antigen in combination with Th1-neutral or Th2-biased adjuvants (e.g., alum, which typically skews toward Th2) to determine whether the Th1 polarization is maintained independently of Freund’s adjuvant.

### Limitations

This study mainly completed the screening of antigens and the identification of immunogenicity. In the present work, in-vivo bacterial challenge protection experiments were not performed, which will be required to verify its actual protective efficacy [12]. In addition, due to the lack of resolution of the protein’s three-dimensional structure, the specific B-cell or T-cell epitopes are still unclear. Furthermore, the use of Freund’s adjuvant, while enabling robust comparative antigen screening, may have confounded the assessment of the intrinsic Th1-polarizing properties of individual antigens; future studies with alternative adjuvants are needed. These will be the focus of subsequent research. Both thepredicted membrane/secreted localization and the apparentabsence of these antigens in the vaccine strain M5 werederived from in silico analyses (SignalP/TMHMMprediction and pangenome orthogroup clustering ofpublicly available draft genome assemblies) and thereforerequire experimental confirmation, as assembly orannotation gaps may yield false absence calls.

## Conclusion

Using a multi-omics joint analysis strategy, this study successfully screened the *Brucella melitensis* candidate antigen BMEI1943. This antigen is highly conserved in virulent strains and can effectively induce a Th1-type immune response marked by IgG2a and IFN-γ in mice. This study provides important candidate molecules for the development of new subunit vaccines against brucellosis and offers new data support for research on the immune mechanism of brucellosis.
