# Correction: integrated multi-omics strategy for screening novel protective antigens against Brucella melitensis and analysis of their immunogenicity

**DOI:** 10.1007/s42770-026-02099-0

**Published:** 2026-10-26

**Authors:** Zhiheng Dong, Sha Li, Jiarong Guo, Lidao Bao

**Affiliations:** 1 Precision Pharmacy Laboratory, The Affiliated Hospital of Inner Mongolia Medical University, Hohhot, 010050 China; 2 Central Laboratory, Hohhot Mongolian Traditional Medicine Hospital, Hohhot, 010050 China

**Correction: Brazilian Journal of Microbiology (2026) 57:244**

**https://doi.org/10.1007/s42770-026-02049-w**

The original online version of this article was revised: 

In the Abstract, Introduction, Methods, and Results sections of this article, references to the chronic infection model involving live *Brucella melitensis* and the associated experimental data should be removed.

In this article, the description of single-cell transcriptome sequencing performed on samples derived from infected animals should be removed, including the corresponding methodological and results descriptions.

In this article, references to P3/BSL-3 containment facilities and to the Inner Mongolia Center for Disease Control and Prevention should be removed.

In this article, the related biosafety and research-conduct statement, ethical statement, conflict-of-interest statement, and author-contribution statement should be removed or amended accordingly.

In this article, the keywords “ABSL-3” and “Single-cell transcriptome” should be removed.

In this article, the title should be amended to reflect the revised scope of the study following removal of the single-cell transcriptome component.

The remaining study components, including comparative genomics and pangenomic analyses, recombinant protein expression and purification, immunization with recombinant proteins and heat-inactivated M5 lysate, ELISA measurements, and flow cytometric detection of intracellular IFN-γ, remain unchanged.

The original article has been corrected.

